# Japan’s Ongoing Crisis on HPV Vaccination

**DOI:** 10.3390/vaccines8030362

**Published:** 2020-07-06

**Authors:** Masayuki Sekine, Risa Kudo, Manako Yamaguchi, Sharon J. B. Hanley, Megumi Hara, Sosuke Adachi, Yutaka Ueda, Etsuko Miyagi, Sayaka Ikeda, Asami Yagi, Takayuki Enomoto

**Affiliations:** 1 Department of Obstetrics and Gynecology, Niigata University Graduate School of Medical and Dental Sciences, Niigata 951-8510, Japan; pearpear@med.niigata-u.ac.jp (R.K.); manako0131@gmail.com (M.Y.); sadachi@med.niigata-u.ac.jp (S.A.); enomoto@med.niigata-u.ac.jp (T.E.); 2 Department of Obstetrics and Gynecology, Hokkaido University Graduate School of Medicine, Sapporo 060-8638, Japan; sjbh1810@mta.biglobe.ne.jp; 3 Department of Preventive Medicine, Faculty of Medicine, Saga University, Saga 849-8501, Japan; harameg@cc.saga-u.ac.jp; 4 Departments of Obstetrics and Gynecology, Osaka University Graduate School of Medicine, Osaka 565-0871, Japan; y.ueda@gyne.med.osaka-u.ac.jp (Y.U.); a.yagi@gyne.med.osaka-u.ac.jp (A.Y.); 5 Department of Obstetrics and Gynecology, Yokohama City University School of Medicine, Yokohama 236-0004, Japan; emiyagi@yokohama-cu.ac.jp; 6 Division of Environmental Medicine and Population Sciences, Department of Social and Environmental Medicine, Graduate School of Medicine, Osaka University, Osaka 565-0871, Japan; sayakaikeda0201@gmail.com

**Keywords:** HPV vaccine, HPV infection, cervical cancer, adverse reaction, Japanese government, suspension of proactive recommendation

## Abstract

The Japanese government suspended proactive recommendations for the HPV vaccine in June 2013. The suspension is now in its seventh year, despite all the data pointing to the safety of the HPV vaccine. We reported a high vaccine effectiveness in the group of women vaccinated before their first intercourse (93.9%). The prevalence of cross-protected types of HPV 31/45/52 was also lower in the vaccinated group, and the vaccine effectiveness was 67.7%. Furthermore, prevalence of HPV16, 31 and 52 infection rates in the vaccinated group were obviously lower than that in the unvaccinated group, and no one had HPV18 or 45 infection in the vaccinated group. The addition of a cross-protective effect toward HPV types 31/45/52 to HPV types 16/18, which is the direct target of the bivalent HPV vaccine, may possibly prevent around 82% of invasive cervical cancer cases in Japan. With regard to the preventive effect of histological abnormalities, we also reported significant reduction in incidence of cervical intraepithelial neoplasia (CIN)3 or worse. Thus, the efficacy of the vaccine has been demonstrated for precancerous disease, and the diverse symptoms after HPV vaccination are likely functional somatic. For the future of Japanese girls, there is a need to resume the proactive recommendation of HPV vaccination and for immediate action to be taken by the Japanese government.

## 1. Introduction

There are more than 10,000 cases of cervical cancer, and nearly 3000 women die from the disease annually in Japan [1]. Incidence of cervical cancer is increasing in young women of reproductive age [2]. A pap smear for cervical cancer screening is recommended for women aged ≥20 years biennially as a secondary prevention. However, the coverage of the screening test is lower than most other Organisation for Economic Co-operation and Development (OECD) countries [3,4]. Therefore, HPV vaccination is particularly important as a secondary prevention for young Japanese women.

The bivalent HPV vaccine was licensed in October 2009, and the quadrivalent vaccine in July 2011. Public funding became available from 2010, and from April 2013, both the bivalent and quadrivalent HPV vaccines were included in the Japanese National Immunization Program (NIP) for girls aged 12–16 years. However, after unfounded sensational reports of so-called ‘adverse events’ were published by the Japanese media, the Japanese Ministry of Health, Labour, and Welfare (MHLW) suspended proactive recommendations for the HPV vaccine in June 2013, only two months after the vaccine had been introduced into the NIP [5]. After that, the vaccine coverage dramatically decreased, from >70% in women eligible for free vaccination between October 2010 and March 2013, when the specially funded program was available, to less than 1% in women eligible for free vaccination after it was introduced into the NIP from April 2013 [5,6]. The Vaccine Adverse Reactions Review Committee (VARRC) of the MHLW investigating these adverse events continues to conclude there is no evidence to suggest a causal association between the HPV vaccine and reported symptoms. However, despite all the data pointing to the safety of the HPV vaccine, the Japanese government continues to suspend its proactive recommendations for it, and the suspension is now in its seventh year. Since incidence of, and mortality from, cervical cancers is increasing in Japanese women of reproductive age and the cancer screening uptake is between 30% and 40% [2], the present situation puts Japanese women at risk of developing a highly preventable cancer [7].

In 2014, the generation of girls who received the HPV vaccination during the first year of its introduction under public funding turned 20 years of age and thereby became eligible for cervical cancer screening. Accordingly, Japanese data on the preventive effects of HPV vaccination against HPV infection and cervical intraepithelial neoplasia (CIN) have been published one after another.

## 2. Efficacy of the HPV Vaccine as a Preventative for Cervical Cancer

### 2.1. Preventive Effect for HPV Infection

We have conducted two studies with different designs to evaluate the efficacy of the HPV vaccine and published the results of the interim analysis [6,8]. This is in reference to the cross-sectional study that enrolled subjects during the population-based cervical cancer screening program (NIIGATA STUDY), and the prospective study that enrolled subjects at the time of vaccination and tracked their cervical cancer screening results at the age of 20 and 25 years (OCEAN STUDY). The strength of both studies is the ability to more accurately distinguish between individuals who were vaccinated and those who were not vaccinated based on the municipal immunization records.

**Figure 1:**
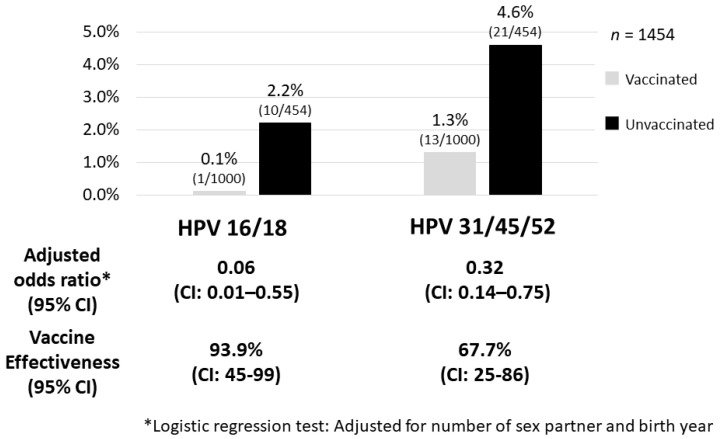
Adjusted vaccine effectiveness against pooled HPV 16/18 and HPV 31/45/52. Whereas the HPV types 16/18 infection rate among the vaccinated group was 0.1%, the infection rate among the unvaccinated group was 2.2%, which indicated a high vaccine effectiveness in the group of women vaccinated before their first intercourse (93.9%). The prevalence of cross-protected types of HPV 31/45/52 was also significantly lower in the vaccinated group where the vaccine effectiveness was 67.7% [6].

**Figure 2:**
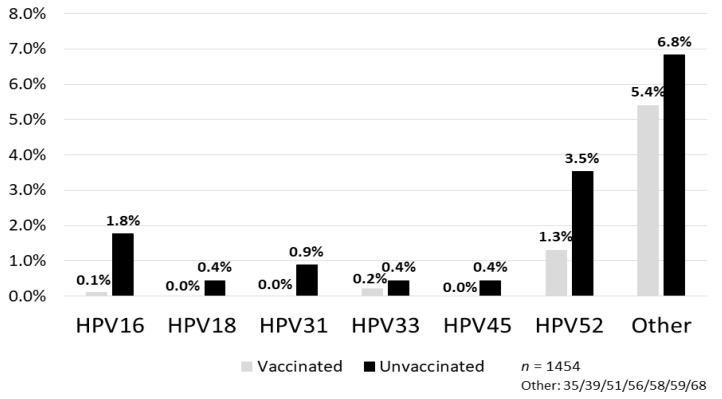
Prevalence of high-risk HPV type-specific infection. Prevalence of HPV16, 31 and 52 infection rates in the vaccinated group were obviously lower than that in the unvaccinated group, and no one had HPV18 or 45 infection in the vaccinated group [6].

The NIIGATA STUDY is a cross-sectional study that examined the effect of the HPV vaccine on the reduction of HPV infections, cytological abnormalities, and histological abnormalities in women who underwent cervical cancer screening in Niigata, Japan [6]. In addition to self-reported vaccination status from questionnaires, we used official municipal immunization records to determine the type of vaccine, vaccination date, and number of vaccination doses received to assign the subjects to either the vaccinated, or the unvaccinated group. In addition, we conducted a questionnaire investigation on the sexual activity of the enrolled women (age at the time of first intercourse, cumulative number of sexual partners, etc.) and calculated the accurate vaccine efficacy that also considered these factors. Women enrolled in the study included 1355 vaccinated individuals (74.6%) with bivalent HPV vaccine and 459 unvaccinated individuals (25.4%). Moreover, 1295 of the vaccinated women (95.5%) had completed three doses of the vaccine. Whereas the HPV types 16/18 infection rate among the vaccinated group was 0.2%, the infection rate among the unvaccinated group was 2.2%, which indicated that the vaccine effectiveness (VE) was 91.9% in preventing HPV types 16/18 infection [6]. Particularly in the group of women vaccinated before their first intercourse, the VE for HPV types 16/18 infection increased further to 93.9% and also prevented HPV types 31/45/52 infections (VE = 67.7%) (Figure 1). A result of additional analysis for the prevalence of high-risk HPV type-specific infections is shown in Figure 2. Prevalence of HPV16, 31 and 52 infection in the vaccinated group was obviously lower than that in the unvaccinated group, and no one had HPV18 or HPV45 infection in the vaccinated group. We have shown cross-protection effects against pooled HR-HPV (high-risk HPV) types 31, 45, and 52, which are associated with an additional 10% of invasive cervical cancer cases in Japan. However, prevalence of other HR-HPV type infections (i.e., HPV 35/39/51/56/58/59/68) in the vaccinated group did not show an obvious decrease compared with the unvaccinated group. If we consider these results, the addition of a cross-protective effect toward HPV types 31/45/52 to HPV types 16/18, which is the direct target of the bivalent HPV vaccine, may possibly prevent around 82% of invasive cervical cancer cases in Japan [9,10]. A nonavalent HPV vaccine, which will be available soon in Japan, provides direct protection against HPV 16/18/31/33/45/52/58, responsible for approximately 90% of cervical cancers in Japan (additional 8% to the effect of the bivalent vaccine).

The OCEAN STUDY is a prospective study that enrolled and tracked individuals who were vaccinated between 12 and 18 years of age, who would undergo HPV testing and cytological and histological examinations at the ages of 20 and 25 years, and compared their results to unvaccinated individuals. The interim analysis showed that in the cervical cancer screening for individuals aged 20 and 21 years, the infection rate of high-risk type HPV was significantly lower in vaccinated individuals at 12.9%, relative to 19.7% in unvaccinated individuals [8]. Particularly, there should be a special mention of the fact that there was not a single case of HPV type 16/18 infection among the vaccinated group.

We have also reported the risks of classifying vaccinated and unvaccinated individuals on the basis of self-reported vaccination status [5]. In young Japanese women, the self-reported memory of HPV vaccination status tends to be inaccurate. Particularly, we raised the major issue where a group of HPV unvaccinated individuals based on self-reports can lead to misclassification of about half of women, given the fact that the negative predictive value (the probability that self-reported unvaccinated individuals actually have not received the HPV vaccine) is approximately 50%. It was a surprising outcome that there were 171 women (11%) who self-reported being unvaccinated, but the actual municipal records revealed that they had, in fact, been vaccinated (thus, these women had forgotten that they had received the HPV vaccine).

### 2.2. Preventive Effect for Cytological Abnormalities

There have been reports on the efficacy of the HPV vaccine using cervical cancer screening data from various regions. Ozawa et al. compared the results of cytological abnormalities in women aged 20–24 years who underwent cervical cancer screening in Miyagi Prefecture and reported that there was a significantly lower prevalence of cytological abnormalities in vaccinated individuals, and the risk of HPV infection was reduced by 52.1%, based on the fact that 2.41% of vaccinated women and 5.03% of unvaccinated women developed cytological abnormalities which were more severe than or equal to ACS-US (ACS-US+) (*p* = 0.03) [11]. Moreover, Tanaka et al. compared the results of cytological abnormalities in women aged 20–24 years who underwent cervical cancer screening in Akita Prefecture and similarly reported a significantly lower prevalence of cytological abnormalities in vaccinated women, and that the VE was 88.1%, based on the fact that 0.24% of vaccinated women and 2.04% of unvaccinated women developed ACS-US+ (*p* = 0.01) [12]. Because of the small number of enrolled women and their young age, these two reports were unable to show any significant differences between vaccinated and unvaccinated women in terms of the prevalence of cytological abnormalities that were more severe than or equal to HSIL (high-grade squamous intraepithelial lesion). The limitation for the abovementioned two studies is the fact that they each classified HPV vaccination status of subjects based on self-reports and did not cross-reference the municipal immunization records.

Organized human papillomavirus vaccination (OHPV) in Japan was introduced in 2010 for girls aged 12–16 years who were born in 1994 or later. The rate of OHPV coverage was 70–80% in girls born from 1994 to 1998. Ueda et al. compared the prevalence of cytological abnormalities in women aged 20 years between two different generations: those from the generation before the start of public funding of the HPV vaccine (pre-OHPV generation: born between 1990 and 1993), and those from the generation in which the vaccination rate increased up to 70% owing to the start of public funding for the vaccine (OHPV generation: born between 1994 and 1995) [13]. There was a significant decrease in the prevalence of ACS-US+ cases in the OHPV generation (3.01%) relative to the pre-OHPV generation (3.96%). Additionally, even when limited to comparison of LSIL+ cases, the OHPV generation showed a prevalence of 0.58% for such abnormalities, which was a significant decrease from the prevalence of 2.11% observed in the pre-OHPV generation.

### 2.3. Preventive Effect for Histological Abnormalities

In the 2018 Japan Cancer Society report by Konno et al. concerning 16 locations nationwide, the prevalence of CIN2+ was significantly lower in vaccinated women and the risk of infection decreased by 69%, based on 0.20% of vaccinated women and 0.66% of unvaccinated women aged between 20 and 29 years with CIN2+ (*p* = 0.01) [14]. Matsumoto et al. conducted an observational study that enrolled female patients aged <40 years in approximately 20 hospitals around Japan who had been diagnosed with a precancerous lesion or cervical cancer to investigate the HPV type 16/18 infection rates and the effect of the HPV vaccine and published the results of the interim analysis using data from 2012 to 2015 [15,16]. A comparison of the positivity rates of HPV type 16/18 infections according to age in the population of patients with CIN1+ showed a decrease from 50.0% to 14.3% in women aged between 20 and 24 years from 2012 to 2015, in contrast to that in the group of patients aged >25 years, who did not show a decreasing trend in the positivity rate. Moreover, investigation of the positivity rates of HPV type 16/18 infections in patients with CIN2+ on the basis of the year of birth showed that patients born between 1986 and 1993, that is, in the pre-OHPV generation, had a positivity rate for infections of 54.6% in contrast to the 23.8% positivity rate of infections among those born between 1994 and 1995, that is, the OHPV generation. As such, there was a significant decrease in the infection rate of HPV type 16/18 infections in the OHPV generation in this report. These two studies have a major limitation in that HPV vaccination status was self-reported by the subjects.

**Figure 3:**
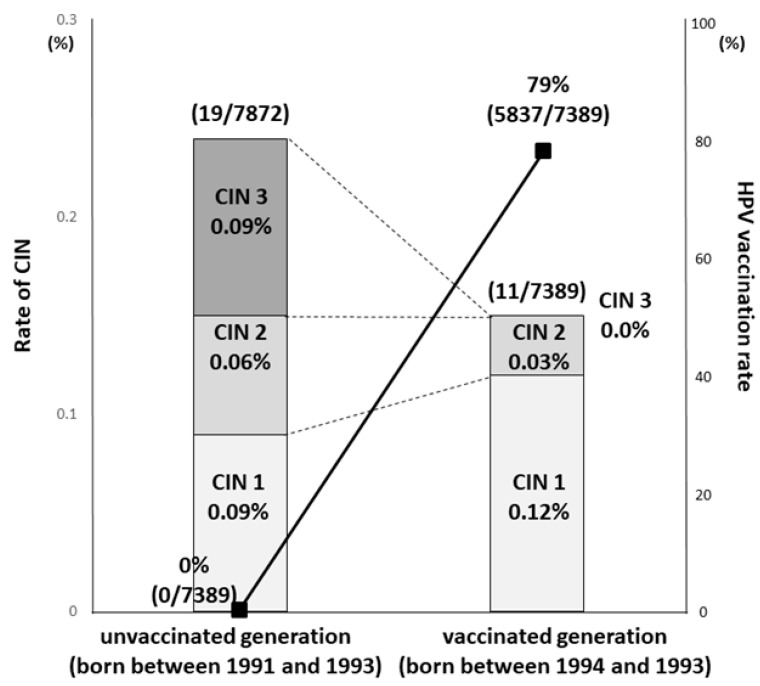
Comparison of the HPV vaccination rate and the results of cervical cancer screening between the unvaccinated and vaccinated generations. The line and bar show HPV vaccination rate and incidence rate of histological abnormalities, respectively. The vaccination rate was 0% (0/7872) in the unvaccinated (born between 1991 and 1993) and 79% (5837/7389) in the vaccinated (born between 1994 and 1996) generations, respectively. The incidence of CIN3 or worse was 0.09% (7/7872) for the unvaccinated generation born between 1991 and 1993, and 0.00% (0/7389) for the vaccinated generation born between 1994 and 1996 [17].

Yagi et al. reported a significant reduction in incidence of CIN3+ depending on birth year in Japan. The incidence of CIN3 or worse was 0.09% (7/7872) for the pre-OHPV generation born between 1991 and 1993, and 0.00% (0/7389) for the OHPV generation born between 1994 and 1996 (Figure 3) [17]. Thus, the efficacy of the HPV vaccine has been demonstrated for precancerous lesions, and we anticipate future results on how well it can prevent cervical cancer.

### 2.4. Impact of Suspension of Proactive Recommendations

If the suspension of proactive recommendations of the HPV vaccine is not lifted and the administration of the vaccine remains halted, we may continue to face a reality wherein the vaccination rates vary significantly according to the year of birth. Whereas women in the OHPV (pre-suspension) generation, born between 1994 and 1999, have a vaccination rate of approximately 70%, among women born after the year 2000, in the post-suspension generation, the vaccination rate reduced to almost 0%. As such, the risk of HPV infection and development of cervical cancer in Japanese women born after 2000 (post-suspension generation) may return to the same state as that in the pre-OHPV generation [18].

## 3. Discussions Related to Safety of the HPV Vaccine

Approximately 80% of women develop localized symptoms, such as temporary pain and inflammation of the injection site, during HPV vaccination. Additionally, there have been reports of cases of women that developed vagal reflex due to pain and anxiety from the injection. The Vaccine Adverse Reactions Review Committee (VARRC) of the MHLW in Japan has expressed its opinion that there has been no evidence relating “The diverse symptoms reported after HPV vaccination” and “HPV vaccine,” and that the symptoms are likely functional somatic [19]. The Cochrane Review also reported that, while there is an increased short-term localized reaction after HPV vaccination, it does not increase the prevalence of serious systemic adverse reactions [20]. The Global Advisory Committee on Vaccine Safety of the WHO (GACVS) has continuously analyzed the latest global data and has expressed their opinion that this vaccine is extremely safe in the latest HPV vaccine safety update [21].

In their national epidemiological study, Sobue et al. reported the following: “It is estimated that among girls between the ages of 12 and 18 years, 40.3 (in a population of 100,000 girls) exhibit “diverse symptoms” after vaccination, similar to those that develop after HPV vaccination, and 20.4 develop “diverse symptoms” without a history of HPV vaccination,” which indicated that a certain number of girls exhibit such “diverse symptoms” even with no history of HPV vaccination [22]. A questionnaire survey independently conducted by Nagoya City also indicated that the frequency of “diverse symptoms,” such as pain, motor disorder, and sleeping disorder, in vaccinated and unvaccinated girls was not significantly different [23]. The results of the abovementioned investigations show that there was no evidence of the association between “HPV vaccination” and “diverse symptoms occurring after vaccination.” Presently, 90 medical institutions around Japan and in all prefectures of Japan have installed a medical consultation window for those who have developed some form of symptoms after receiving HPV vaccination [24], but the HPV vaccination rate remains at nearly 0% in Japan.

## 4. Prospects for the Future

Australia has published a report indicating that cervical cancer can be eradicated and has proposed a timeline toward its realization [25]. They estimated that if a system can be put in place for girls aged 12–15 years to receive the HPV vaccine and undergo tests to check for the presence of HPV infection just twice over the course of their lifetime, it would be possible to reduce the number of patients with cervical cancer by 1.3 million over the course of 50 years, and by the end of this century, there will be <4 patients per 100,000 individuals in most countries. This was covered widely in the media, which also shed light on Japan’s gloomy future regarding the disease [26]. Although HPV vaccines were included in the Japanese NIP, it has been 7 years since the proactive recommendation of HPV vaccination was suspended. Even if proactive recommendation of the vaccine is currently resumed, women born after the year 2000 have already surpassed the age indicated for HPV vaccination (12–16 years) and will not be able to receive the benefits of such a routine national vaccination. There is no Japanese male received HPV vaccination at public expense, because the Japanese NIP has not included men. Given the increasing incidence of oropharyngeal cancer in men, it is important to discuss the issue of HPV vaccination for men. However, it seems very difficult to include men in the NIP in the current situation of Japan, where almost all Japanese women do not wish to have the vaccination due to “diverse symptoms” after vaccination.

The MHLW in Japan approved the manufacturing and marketing of the nonavalent vaccine on 20 May 2020. In Japan, the application for the vaccine was submitted on 3 July 2015. However, the approval reviews took an unusually long period of about five years. After approval by the MHLW, further discussions will be conducted in the subcommittee for use in NIP. For the nonavalent vaccine, the Japanese Society of Obstetrics and Gynecology has repeatedly requested that the Japanese government obtain early approval for a routine immunization program. A strong opposition movement against HPV vaccination and lawsuits against the Japanese government and pharmaceutical companies seem to be the cause of the delay in approval. The nonavalent HPV vaccine may be able to prevent approximately 90% of all HPV-related cervical cancer cases [26] and is a promising vaccine for a country like Japan, where the involvement of high-risk non-16/18 type HPV infections in cervical cancer development is higher than that in Western countries, which is why there is a need to resume the proactive recommendation of HPV vaccination and immediate action to be taken by the Japanese government.

## References

[1] Cancer Information Service, National Cancer Center, Japan Cancer Registry and Statistics. https://ganjoho.jp/reg_stat/statistics/dl/.

[2] Motoki Y., Mizushima S., Taguri M., Takahashi K., Asano R., Kato H., Asai-Sato M., Katayama K., Okamoto N., Hirahara F. (2015). Increasing trends in cervical cancer mortality among young Japanese women below the age of 50 years: An analysis using the kanagawa population-based cancer registry, 1975–2012. Cancer Epidemiol..

[3] Sauvaget C., Nishino Y., Konno R., Tase T., Morimoto T., Hisamichi S. (2016). Challenges in breast and cervical cancer control in Japan. Lancet Oncol..

[4] OECD Stat Health Care Utilisation: Screening. https://stats.oecd.org/index.aspx?queryid=30159.

[5] Yamaguchi M., Sekine M., Kudo R., Adachi S., Ueda Y., Miyagi E., Hara M., Hanley S.J., Enomoto T. (2018). Differential misclassification between self-reported status and official HPV vaccination records in Japan: Implications for evaluating vaccine safety and effectiveness. Papillomavirus Res. (Amst. Neth.).

[6] Kudo R., Yamaguchi M., Sekine M., Adachi S., Ueda Y., Miyagi E., Hara M., Hanley S.J.B., Enomoto T. (2018). Bivalent Human Papillomavirus Vaccine Effectiveness in a Japanese Population: High Vaccine-Type–Specific Effectiveness and Evidence of Cross-Protection. J. Infect. Dis..

[7] Yagi A., Ueda Y., Egawa-Takata T., Tanaka Y., Nakae R., Morimoto A., Terai Y., Ohmichi M., Ichimura T., Sumi T. (2017). Realistic fear of cervical cancer risk in Japan depending on birth year. Hum. Vaccines Immunother..

[8] Japan Society of Obstetrics and Gynecology: For the Current Knowledge and Correct Understanding of Cervical Cancer and HPV Vaccine. https://www.jsog.or.jp/uploads/files/jsogpolicy/HPV_Q%26A.pdf.

[9] Azuma Y., Kusumoto-Matsuo R., Takeuchi F., Uenoyama A., Kondo K., Tsunoda H., Nagasaka K., Kawana K., Morisada T., Iwata T. (2014). Human papillomavirus genotype distribution in cervical intraepithelial neoplasia grade 2/3 and invasive cervical cancer in Japanese women. Jpn. J. Clin. Oncol..

[10] Matsumoto K., Yoshikawa H. (2012). Human papillomavirus infection and the risk of cervical cancer in Japan. J. Obstet. Gynaecol. Res..

[11] Ozawa N., Ito K., Tase T., Metoki H., Yaegashi N. (2016). Beneficial Effects of Human Papillomavirus Vaccine for Prevention of Cervical Abnormalities in Miyagi, Japan. Tohoku J. Exp. Med..

[12] Tanaka H., Shirasawa H., Shimizu D., Sato N., Ooyama N., Takahashi O., Terada Y. (2017). Preventive effect of human papillomavirus vaccination on the development of uterine cervical lesions in young Japanese women. J. Obstet. Gynaecol. Res..

[13] Ueda Y., Yagi A., Nakayama T., Hirai K., Ikeda S., Sekine M., Miyagi E., Enomoto T. (2018). Dynamic changes in Japan’s prevalence of abnormal findings in cervical cervical cytology depending on birth year. Sci. Rep..

[14] Konno R., Konishi H., Sauvaget C., Ohashi Y., Kakizoe T. (2018). Effectiveness of HPV vaccination against high grade cervical lesions in Japan. Vaccine.

[15] Matsumoto K., Yaegashi N., Iwata T., Ariyoshi K., Fujiwara K., Shiroyama Y., Usami T., Kawano Y., Horie K., Kawano K. (2014). Monitoring the Impact of a National HPV Vaccination Program in Japan (MINT Study): Rationale, Design and Methods. Jpn. J. Clin. Oncol..

[16] Matsumoto K., Yaegashi N., Iwata T., Yamamoto K., Nagashima M., Saito T., Ushijima K., Takahashi F., Noda K., Yoshikawa H. (2017). Early impact of the Japanese immunization program implemented before the HPV vaccination crisis. Int. J. Cancer.

[17] Yagi A., Ueda Y., Ikeda S., Sekine M., Nakayama T., Miyagi E., Enomoto T. (2019). Evaluation of future cervical cancer risk in Japan, based on birth year. Vaccine.

[18] Tanaka Y., Ueda Y., Egawa-Takata T., Yagi A., Yoshino K., Kimura T. (2016). Outcomes for girls without HPV vaccination in Japan. Lancet Oncol..

[19] The 15th Vaccination Sub-committee Meeting: Investigative Committee on Adverse Reactions, Health Sciences Council, Ministry of Health, Labour, and Welfare (17 September 2015). https://www.mhlw.go.jp/stf/shingi2/0000097690.html.

[20] Arbyn M., Xu L. (2018). Efficacy and safety of prophylactic HPV vaccines. A Cochrane review of randomized trials. Expert Rev. Vaccines.

[21] WHO Draft Global Strategy towards the Elimination of Cervical Cancer as a Public Health Problem. http://www.jsog.or.jp/uploads/files/jsogpolicy/WHO-slides_CxCaElimination.pdf.

[22] The 23th Vaccination Sub-Committee Meeting: Investigative Committee on Adverse Reactions, Health Sciences Council, Ministry of Health, Labour, and Welfare (26 December 2016). https://www.mhlw.go.jp/stf/shingi2/0000150170.html.

[23] Suzuki S., Hosono A. (2018). No association between HPV vaccine and reported post-vaccination symptoms in Japanese young women: Results of the Nagoya study. Papillomavirus Res. (Amst. Neth.).

[24] Medical Institutions Involved in the Treatment of Symptoms that Occurred after HPV Vaccination. https://www.mhlw.go.jp/bunya/kenkou/kekkaku-kansenshou28/medical_institution/dl/kyoyroku.pdf.

[25] Simms K.T., Steinberg J., Caruana M., Smith M.A., Lew J.B., Sperjomataram I., Castle P.E., Bray F., Canfell K. (2019). Impact of scaled up human papillomavirus vaccination and cervical screening and the potential for global elimination of cervical cancer in 181 countries, 2020-99: A modelling study. Lancet Oncol..

[26] Chatterjee A. (2014). The next generation of HPV vaccines: Nonavalent vaccine V503 on the horizon. Expert Rev. Vaccines.

